# Analysis of risk factor for nail breakage in patients with mechanical failures after proximal femoral nail antirotation in intertrochanteric fractures

**DOI:** 10.1097/MD.0000000000029436

**Published:** 2022-06-24

**Authors:** Young-Kyun Lee, Jung-Taek Kim, Chan Ho Park, Ji-Ung Song, Tae-Young Kim, Kyung-Hoi Koo

**Affiliations:** a Department of Orthopedic Surgery, Seoul National University Bundang Hospital, Seongnam, Korea; b Department of Orthopedic Surgery, Ajou Medical Center, Ajou University School of Medicine, Suwon, Korea; c Department of Orthopedic Surgery, Yeungnam University Medical Center, Daegu, Korea; d Department of Orthopedic Surgery, Chamjoeun Hospital, Gwangju, Korea; e Department of Orthopedic Surgery, Konkuk University Medical Center, Seoul, Korea.

**Keywords:** horizontal offset, intertrochanteric fracture, nail breakage

## Abstract

Breakage of the intramedullary nail is a rare complication after proximal femoral nail antirotation (PFNA) in intertrochanteric fracture treatment. The purpose of this study was (1) to investigate the frequency of nail breakage among the patients who were treated for mechanical failure after PFNA for intertrochanteric/pertrochanteric fracture, and (2) to determine the risk factors for nail breakage in PFNA treatment of intertrochanteric fracture.

To identify mechanical failure after internal fixation using PFNA, we retrospectively reviewed the data of 35 patients (35 hips) who required reoperation after PFNA with a helical blade for intertrochanteric/pertrochanteric fracture between June 2005 and June 2018.

We evaluated the frequency of breakage of PFNA and compared the demographic and radiologic parameters between the breakage and control (non-breakage) groups. We also compared the lever arm for the load of stress from the fulcrum according to the centrum-collum-diaphyseal (CCD) angle of blade by using reverse design technique.

Among the 25 patients with mechanical failure after PFNA except 10 patients with peri-implant infection and osteonecrosis, 7 (28.0%) showed breakage of PFNA at average of 8 months (range, 5–13 months) after index surgery. A larger horizontal offset (the horizontal distance from the lateral surface of the IM nail and the medial tip of helical blade) was associated with an increased risk of nail breakage. A CCD angle of 130° has a shorter lever arm for the load of stress from the fulcrum, meaning a higher stress for nail breakage, although there was no association between CCD angle and breakage of the nail.

Our study suggested that higher horizontal offset and a higher CCD angle can increase the risk of breakage of the PFNA nail at the aperture for the helical blade.

## 1 Introduction

The treatment of intertrochanteric fracture is a significant challenge.^[1]^ Traditionally, internal fixation by treatment with an extramedullary plate and sliding hip screw has been the preferred treatment option for this type of fracture.^[2]^ Since the intramedullary nail was introduced in 1990s, it has become increasingly popular globally over time.^[3–5]^

Several types of cephalomedullary nails are commercially available; proximal femoral nail antirotation (PFNA; Synthes, Solothurn, Switzerland) is the most advanced version featuring an anti-rotation helical blade from the proximal femoral nail.^[6]^ Cephalomedullary nails have been reported to have advantages in biomechanical stability when compared to extramedullary plates with a sliding hip screw for the treatment of intertrochanteric fracture.^[7–9]^ However, the PFNA system has been shown to be prone to mechanical failures, with a failure rate of 2.6–13% in patients receiving PFNA.^[10]^ Mechanical failures recorded include non-union, cut-out or cut-through, migration of the screw or blade, peri-implant fracture, and breakage of the implant.^[11]^

To avoid mechanical failure such as non-union, cut-out or cut-through, and migration of the screw or blade, surgeons try to appropriately modify the adjustable factors, including the reduction state, position of the lag screw, tip-apex index, and entry point of the nail. These precautions are to reduce implant-related complications during PFNA.^[10,12]^ However, to date, there are few studies on the frequency and affecting factors of implant breakage after PFNA nailing of intertrochanteric fractures.

Therefore, the purpose of this study was (1) to evaluate the frequency of implant breakage among the patients who were treated for mechanical failure after PFNA for intertrochanteric/pertrochanteric fracture and (2) to determine the associated factors with breakage of PFNA.

## 2 Material and methods

We performed a retrospective multicenter study. The present study protocol was reviewed and approved by the Institutional Review Board of Seoul National University Bundang Hospital (approval No. B-1907/555-107) at June 24, 2020. The need to obtain informed consent was waived because of the retrospective nature of the study.

The inclusion criterion was reoperation for mechanical complications after treatment with the PFNA System for intertrochanteric/pertrochanteric fracture. From June 2005 and June 2018 at 5 tertiary referral hospitals, 35 patients underwent reoperation because of treatment failure. We reviewed the medical records and radiographs of 35 patients (35 hips). Among them, 10 patients underwent reoperation due to problems not related to mechanical complications, such as surgical site infection or osteonecrosis, and thus were excluded from the study.

The remaining 25 patients (of whom 15 underwent the index surgery performed at one of the 5 participating centers, and 10 underwent the index surgery elsewhere) were included for evaluation in this retrospective study. Because the index surgeries were not performed exclusively at the 5 participating centers, we cannot comment on the indications for implant selection in the index surgery [such as why some patients may have received PFNA with a centrum-collum-diaphyseal (CCD) angle of 125° or 130°].

**Figure 1:**
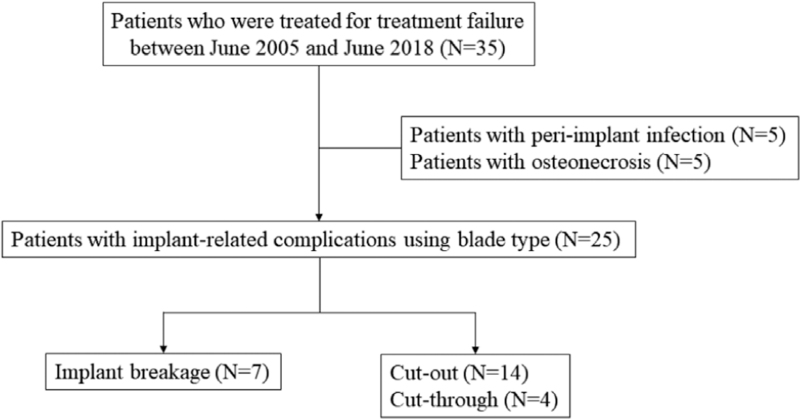
Flow chart to identify patients who met the inclusion criteria for this study.

25 patients with mechanical complications were divided into two groups: group 1 (broken nail) and group 2 (cut through or cut out). The reasons for reoperations were cutting out in 14 patients and cutting through in 4 patients. The remaining 7 patients had reoperation due to a broken PFNA at the level of the hole for the blade (Fig. 1).

Of the included 25 patients, there were 9 men and 16 women, and the mean age at time of index surgery was 75.7 ± 10.56 years (range 45.2–94.0 years). Reoperations were performed at a mean of 5.4 months (range, 0.4–13 months) after the index operation. For the index surgery, PFNA with a CCD angle of 125° was used in 13 patients and that with a CCD angle of 130° was used in 12 patients (Table 1).

**Table 1:** Demographic data of patients in the two groups.

Characteristics	125°(n = 12)	130°(n = 13)	*P*
Gender			
Male	4	4	1.000
Female	8	8	
Age (years)	74.2 ± 11.13	77.8 ± 9.70	.387
BMI (kg/m^2^)	22.8 ± 4.74	24.1 ± 3.11	.403
ASA class	2.2 ± 0.55	2.3 ± 0.63	.515
Anesthesia			
General	7	6	.561
Spinal	5	7	
Interval between index surgery and reoperation (months)	6.2 ± 4.76	4.6 ± 4.30	.377

ASA = American Society of Anesthesiologists, BMI = body mass index.

**Figure 2:**
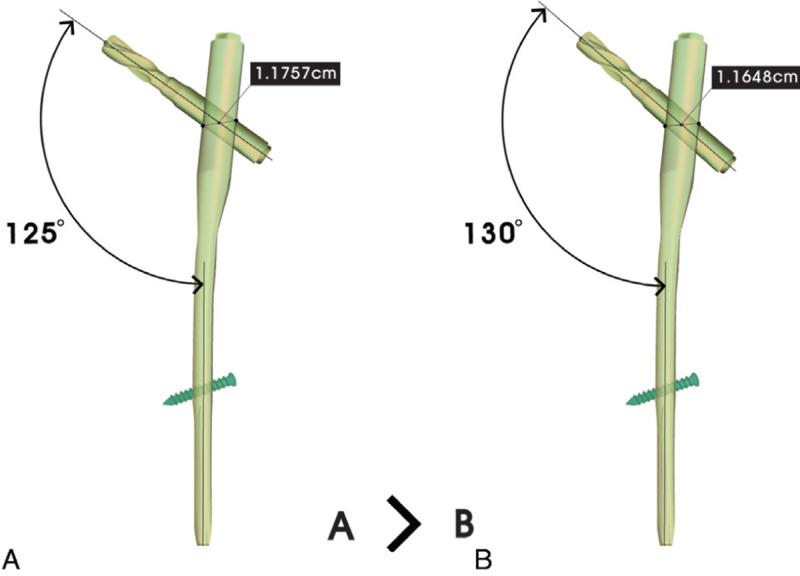
Reverse design technique showed that the lever arm distance from fulcrum to the load of stress (the lateral aperture for the blade) in CCD angle of 130° is shorter than CCD angle of 125°. CCD = centrum-collum-diaphyseal.

To determine the associated risk factors for breakage of PFNA, we compared patient characteristics including age, sex, body mass index, the American Society of Anesthesiologists classification,^[13]^ the CCD angle, and the horizontal offset of the used blade between the broken and non-broken groups. The CCD angle of the PFNA was categorized as either 125° or 130° (Fig. 2).

**Figure 3:**
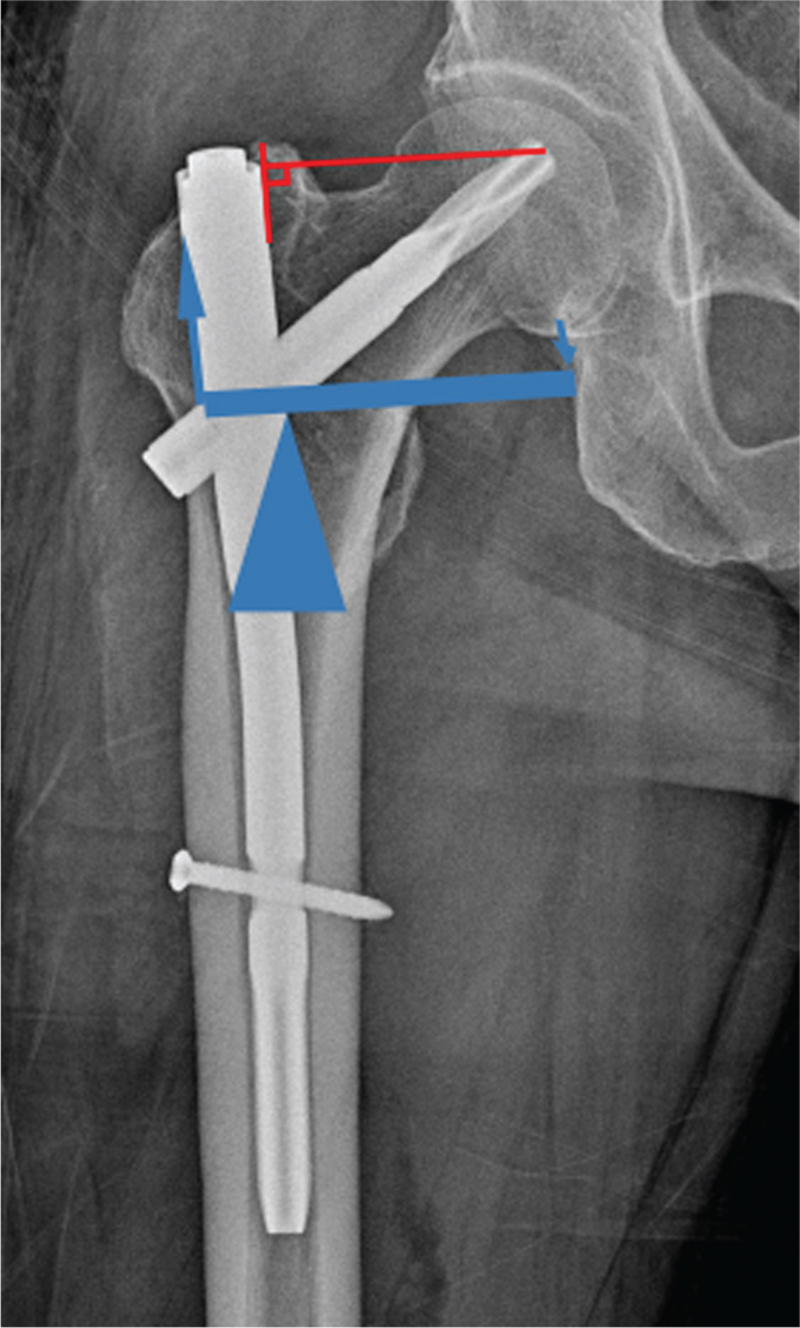
Horizontal offset was defined as the horizontal distance between the medial surface of the intramedullary nail and the medial tip of helical blade. Horizontal offset is the lever arm of first class lever on schematic drawing.

Horizontal offset was defined as the horizontal distance from the lateral surface of the intramedullary nail and the medial tip of helical blade (Fig. 3). The horizontal offset was calculated by correcting magnification using the known diameter of the implanted helical blade.^[14]^

The fracture reduction quality by Fogagnolo et al^[15]^ and the tip-apex distance (TAD)^[14]^ could not be compared, because immediate postoperative radiographs were not available in some patients who underwent index surgery elsewhere.

Statistical analyses were performed with univariate comparisons using Mann-Whitney U test for continuous variables and Fisher's exact test for categorized data. Then, multivariable logistic regression analyses were performed for continuous variables. Differences were considered significant if *p* values were <.05. All analyses were performed using SPSS version 20.0 for Windows (SPSS Inc., Chicago, IL).

## 3 Results

**Figure 4:**
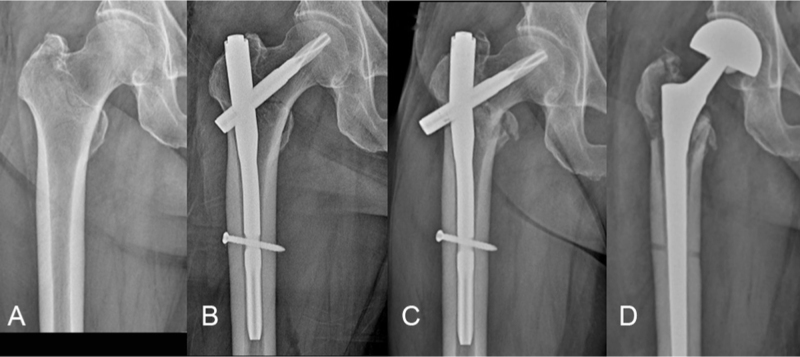
(A) A 73-year-old man who had an intertrochanteric fracture (B) The patient underwent cephalomedullary fixation using PFNA of 130° centrum-collum-diaphyseal angle (C) After 5 months, implant breakage were found (D) Conversion to bipolar hemiarthroplasty was performed.

Seven patients (28.0%) showed breakage of PFNA at average of 8 months (range, 5–13 months) after index surgery among 25 patients who had mechanical complications. All implant breakages went through the proximal aperture for the helical blade. In the broken PFNA group, conversion to hip arthroplasty was performed in 3 patients and osteosynthesis operation in 3 patients (Fig. 4). Another patient was treated conservatively because the patient refused to undergo reoperation.

In the non-broken group, conversion to hip arthroplasty was performed in 17 patients and lag screw removal was performed in 1 patient because the patient refused the hip arthroplasty.

In the univariate analysis, horizontal offset, weight, and height in the broken group were significantly higher than those in the control group; however, the age, sex, body mass index, American Society of Anesthesiologists, type of anesthesia, and CCD angle were not significantly different between both groups (Table 2). Multivariable logistic regression analyses, including those for height, weight, and horizontal offset, showed no statistical significance between both groups.

**Table 2:** Comparison between the broken and control groups.

Characteristics	Broken group(n = 7)	Control group(n = 18)	*P*
Age (years)	73.6 ± 7.1	76.6 ± 11.7	.530
Gender			
Male	4	5	.205
Female	3	13	
Height (cm)	166.1 ± 5.6	156.0 ± 7.9	.008
Weight (kg)	64.9 ± 13.61	54.7 ± 9.7	.004
BMI (kg/m^2^)	25.1 ± 3.08	22.6 ± 4.20	.168
ASA class	2.1 ± 0.69	2.3 ± 0.58	.135
Anesthesia			
General	2	12	.083
Spinal	5	6	
CCD angle			
125°	2	11	.202
130°	5	7	
Horizontal offset (mm)	54.0 ± 5.2	44.8 ± 6.1	.005

ASA = American Society of Anesthesiologists, BMI = body mass index, CCD = centrum-collum-diaphyseal.

## 4 Discussion

Implant breakage is an uncommon complication of patients treated with cephalomedullary nails for intertrochanteric/pertrochanteric fractures. However, some studies have reported breakages of another type of nail such as the Gamma nail (Stryker, Mahwah, NJ) for proximal hip fractures, including subtrochanteric fracture.^[16–20]^ However, until now there have been no studies investigating the breakage of PFNA implants for intertrochanteric/pertrochanteric fractures. To the best of our knowledge, this is the first study to report the frequency of breakage after PFNA for intertrochanteric/pertrochanteric fracture. We found that the overall frequency of implant breakages after PFNA for intertrochanteric/pertrochanteic fracture was 28.0%, among patients with mechanical complications and failure.

Moreover, a longer horizontal offset of the blade was associated with breakage of the PFNA nail. All breakages of the nail went through the proximal aperture for the helical blade. We can hypothesize that the horizontal offset of the helical blade can influence breakage of the PFNA nail. Stress forces on the medial tip of helical blade made it act as a first-class lever. The lower margin of the medial aperture of the blade then acts as the fulcrum. This applies a tensile stress/load to the upper margin of the lateral aperture of the blade. Therefore, a longer horizontal offset of the blade creates a longer lever arm of effect and can increase the tensile stress at the lateral aperture of the blade. Certain conditions can enlarge the horizontal offset of the blade. It might be a good example that distraction of the fracture site can require longer blade for adequate tip apex distance.

Considering the first-class lever action, the distance between the fulcrum and the lateral aperture of the blade can also influence the stress at the lateral aperture of the blade. The shorter lever arm for the load will increase the stress forces at the lateral surface.

The manufacturer does not provide any exact information on the distance between the fulcrum (the lower margin of medial aperture for blade) and the point of load (the upper margin of lateral aperture for the blade). We calculated the distance through reverse design technique using the limited available information that the manufacturer provides to the public (the proximal diameter of 16.5 mm and diameter of helical blade of 11.5 mm). Through this reverse design technique, we could estimate the distance of the lever arm to determine that the load point is 1.1757 cm in the 125° nail and 1.1648 cm in the 130° nail (Fig. 2).

Our reverse design technique suggested that a PFNA with a CCD of 130° has a shorter lever arm for load than that of the 125° PFNA. This means that a CCD of 130° has a higher risk of breakage; however, there was no association between the CCD angle and breakage of the nail in our study. The small sample number of events (breakages) could be a reason for the lack of statistical significance between CCD angles in our study. When the horizontal offset of the blade was fixed or constant to obtain optimal fixation with adequate TAD, PFNA with a CCD of 130° has a shorter lever arm of load, which results in increased stress and a higher risk of breakage in the case of nonunion events. A previous finite element analysis study showed that a higher CCD angle had larger stress in the direction of the sliding blade and it induced greater medial rotation of the proximal fragment.^[21]^

To overcome the risk of breakage of the nail, the manufacturer has recently developed a new design of nail. This new nail encompassed the improvement of not only the mechanical properties but also the design of the device. This new device named the TFN-ADVANCED system (TFNA, Synthes, Solothurn, Switzerland) has been introduced. According to the manufacturer, the TFNA nail consists of a higher strength titanium alloy, thereby improving its mechanical properties, and has a different design of the aperture for the blade named as having a “bump cut design” to increase resistance of the mechanical stresses at the hole. However, there was a report of 16 implant breakages of TFNA in 13 patients. The study suggested cautious surveillance of patients with unstable hip fracture who were treated with a TFNA implant.^[22]^ They did not evaluate the CCD angle of the broken nails.

There were several limitations in this study. First, our study was retrospective, and the number of patients was too small to determine associated factors. However, the breakage of fixation devices is a rare event. Considering this rarity of breakage, a well-designed, larger, multicenter study will be needed in the future to improve on our findings. Second, we could not evaluate other factors including fracture type, reduction quality, the TAD and position of blade at the immediate postoperative, because many patients underwent the index surgery elsewhere in this retrospective study. For example, intertrochanteric fractures, poor reduction, larger TAD and anterosuperior position of blade have been known to be more prone to complications like implant breakage. Third, we did not use the real distance between the fulcrum and the load point, because the manufacturer did not provide this information. However, we used the reverse design technique to obtain an estimated real distance, by using the available information that the manufacturer reveals to the public. Reverse design technique is useful and valid method in this situation. Fourth, we calculated the distance of the lever arm of the load just in the coronal plane. Stress forces act as both torsion and tension; therefore, the stress forces would need to be investigated in other planes.

Despite the limitations, our results showed the frequency of implant breakage after PFNA for intertrochanteric/pertrochanteric fracture. When combined with the results of previous finite element method studies, we noticed that a higher horizontal offset and a higher CCD angle can increase the risk of breakage of the PFNA nail at the aperture for the helical blade.

## Author contributions

Conceptualization: YK Lee Methodology: JT Kim, JU Song Formal analysis: JT Kim, TY Kim Original draft preparation: CH Park Review and editing: YK Lee, KH Koo Approval of final manuscript: all authors.

**Funding acquisition:** Chan Ho Park.

**Writing – original draft:** Chan Ho Park.
